# Predictive factors for Day 7 positive patch test readings at a secondary referral centre

**DOI:** 10.1002/ski2.79

**Published:** 2021-11-24

**Authors:** R. A. Tupker, W. G. C. Stapper, J. C. Kelder

**Affiliations:** ^1^ Dermatology Department St. Antonius Hospital Nieuwegein The Netherlands; ^2^ Epidemiology Department St. Antonius Hospital Nieuwegein The Netherlands

## Abstract

**Background:**

Based on studies at tertiary centres it is known that patch test reading on Day (D) 7 may show additional positive reactions. Female gender, higher age and allergen groups of topicals and corticosteroids were identified as predictive factors.

**Objectives:**

The first aim was to study the value of reading patch tests on D2, D3 and D7 at a secondary referral centre. The second aim was to investigate the predictive potential of the factors sex, age, atopic dermatitis, body location, allergen group and clinical relevance for a positive reaction only on D7.

**Methods:**

Retrospective data from patients tested between 2013 and 2016 were evaluated. The factors sex, age, atopic dermatitis, body location, allergen group and clinical relevance were tested by regression analysis.

**Results:**

Two hundred and sixty‐three out of a total of 396 patients had a positive reaction only on D2, D3 and D7 in 14 (2.5%), 152 (27.5%) and 61 (11.0%) occasions, observed in 10 (2.5%), 108 (27.3%) and 51 (12.9%) patients, respectively. These reactions were deemed relevant in 0 (0%), 12 (2.2%) and 9 (1.6%) occasions, observed 0 (0%), 11 (2.8%) and 9 (2.3%) patients, respectively. Higher age and allergen groups of metals, fragrances and resins were predictive for late positive reactions.

**Conclusions:**

D7 patch test reading should also be routinely adopted at secondary referral centres. D7 positive reactions were associated with higher age and sensitization to metals, fragrances and resins.

**What's already known about this topic?**

- Late patch test reading on Day 7 can identify new positive patch test reactions which were negative on preceding readings. This finding is derived from tertiary referral centres.
- Predicting factors for positive readings on Day 7 are female gender, higher age and allergen groups of topicals and corticosteroids.

**What does this study add?**

- Day 7 patch test reading should also be routinely adopted at secondary referral centres. Day 2 reading may be omitted on strict conditions.
- Predicting factors for Day 7 positive reactions were higher age and allergen groups of metals, fragrances and resins.

## 1 INTRODUCTION

Allergic contact dermatitis (ACD) is caused by delayed‐type allergy (type IV hypersensitivity) to contact allergens. The pathogenesis of ACD involves an initial sensitization phase when the patient first comes in contact with the chemical. In the elicitation phase re‐exposure of the same chemical to the primed T‐cell milieu causes release of cytokines resulting in the clinical picture of ACD. When contact allergy is suspected patch testing should be performed. It is recommended that at least two readings of the test reactions are performed. Ideally, readings are done at Day (D) 2, D3 or D4, and around D7.^1^ However, in practice many dermatologists skip the late reading on D7 because it is time‐consuming both for the patient and the out‐patient staff. Instead, readings are performed only on D2 and D3/D4.

The value of D7 reading is based on studies at tertiary referral centres.^2^, ^3^, ^4^, ^5^, ^6^, ^7^, ^8^, ^9^ Contact allergens most notably causing late reactions were corticosteroids, metals and topicals such as neomycin.^2^, ^3^, ^4^, ^5^, ^6^, ^7^, ^8^, ^9^ The first aim was to study the value of an additional D7 reading at a large secondary referral centre. The fact that the population in secondary referral clinics is less preselected may influence the patch test outcomes.^10^ Apart from scoring the reactions on D2, D3 and D7, their clinical relevance for the patient's dermatitis was estimated in order to optimize the decision on omitting one particular reading day.

To our knowledge, two studies addressed the issue of factors that may contribute to the development of D7 positive reactions.^2^, ^3^ Female gender,^2^ higher age,^2^, ^3^ and allergen groups of topicals and corticosteroids^3^ were identified as factors that can predict late reactions. The second aim of this study was to investigate which factors may be associated with D7 positive patch test results. The factors sex, age, past or current atopic dermatitis, body location, allergen group and clinical relevance were tested.

## 2 PATIENTS AND METHODS

### 2.1 Study design and patch testing

In this retrospective study, adult patients were patch tested between March 2013 and June 2016 at the Dermatology Department of St. Antonius Hospital in Nieuwegein and Utrecht, the Netherlands.

Patients were tested with the extended European Baseline Series as proposed by the Dutch guidelines on contact dermatitis.^11^ The 38 allergens of this series were purchased from Chemotechnique (Chemotechnique MB Diagnostics AB).

Patch test materials were applied on the back of the patient using Van der Bend square Chambers^®^ (Van der Bend) and Fixomull^®^ tape (Beiersdorf) on D0 (Mondays or Tuesdays) and removed on D2. From January 2016, methylisothiazolinone was added to the test series. Readings were carried out on D2 (Wednesdays or Thursdays), D3 (Thursdays or Fridays) and D7 (Mondays or Tuesdays). The readings were done according to the recommendations by the European Society of Contact Dermatitis^1^ which meant that reactions classified as +, ++ or +++ were reported as positive. Doubtful (?+) and irritant reactions were considered negative. A positive reaction on D2, which became negative on D3 and D7 was defined as an ‘isolated’ D2 (‘early’) reaction. A reaction that was positive only on D3 or D7 was defined as an isolated D3 reaction or D7 (‘late’) reaction. A positive patch test reaction was considered clinically relevant if the presence of the current dermatitis was explained by exposure to the allergen concerned.^1^ Possible outcomes were ‘current relevance’ and ‘unknown or past relevance’. It was not possible to make the subdivision into ‘past’ and ‘unknown’ relevance, because this was not always mentioned in the patient files.

### 2.2 Data analysis

The timing of positive reactions was categorized according to the permutation scheme, 0‐0‐0, 0‐0‐1, 0‐1‐0, 1‐0‐0, 0‐1‐1, 1‐0‐1, 1‐1‐0 and 1‐1‐1 (see Table S1). The digits represent the value of the reading on D2, D3 and D7, respectively. The value ‘0’ refers to ‘no positive reaction’, and the value ‘1’ to ‘positive reaction’ on that particular day. The patient characteristics that were analysed were sex, age tested categorized (<18, 19–30, 31–45, 46–60, >60 years) and as a continuous variable, past or current atopic dermatitis, main body location (head [face‐neck‐scalp], hands, feet, other location), and allergens. Allergens were clustered in eight groups: metals, preservatives, fragrances, rubbers, dyes, topical agents, corticosteroids and resins (see Table S2).

### 2.3 Statistics

Standard statistics were used for the descriptive analyses. Generalized linear mixed models (GLMM) regression was used to assess risk factors for an isolated D7 positive skin reaction. GLMM allows for one patient to have multiple readings. Patients were modelled as random effect by means of a random intercept. The regression analyses is performed at two hierarchical levels: at patient level and within patients at patch test reaction level. Showing the results at patch test reaction level was the only way to overcome the fact that one patient could have more positive reactions per allergen group or over more allergen groups on one particular day or on several days.

For multivariable regression analysis we reduced the allergen groups with eight categories post‐hoc into six categories: metals, fragrances, resins, topicals, corticosteroids and others.

For measure of effect, we report odds ratios (OR) and 95% confidence intervals (CI). For the computations the statistical software environment R^12^ was used (version 4 with the ‘lme4’ package^13^).

## 3 RESULTS

### 3.1 Occurrence of isolated D7 reaction

Initially, 623 adult patients were patch tested. Patients who were unable to attend for one or more readings on D2, D3 and D7 were excluded from the study, leaving 396 patients for further analysis. The total number of positive reactions was 553 observed in 263 out of the 396 patients.

Sixty patients were tested with methylisothiazolinone. The majority of the positive reactions (129 reactions [23.3%]) were positive on all 3 days. Isolated positive reactions were noted on D2, D3 and D7 in 14 (2.5%), 152 (27.5%) and 61 (11.0%) occasions, observed in 10 (2.5%), 108 (27.3%) and 51 (12.9%) patients, respectively.

The highest proportions of isolated D7 reactions were observed in the allergen groups topicals, corticosteroids and resins (Table 1). For absolute numbers, the groups of allergens with the highest scores were metals, fragrances and resins. Data on reaction patterns of individual allergens are mentioned in Table S3.

**TABLE 1:** Patch test results for the allergen groups on Days 2, 3 and 7 according to the permutation scheme

Allergen group	0‐0‐0^a^	0‐0‐1	0‐1‐0	1‐0‐0	0‐1‐1	1‐0‐1	1‐1‐0	1‐1‐1	Day 2, 3, 7
Metals	990	25 (13)	37 (19)	3 (2)	55 (28)	0 (0)	19 (10)	59 (30)	198 (100)
Preservatives	3870	7 (8)^b^	37 (42)	2 (2)	23 (26)	0 (0)	7 (8)	12 (14)	88 (100)
Fragrances	1445	10 (7)	48 (35)	4 (3)	41 (29)	1 (1)	8 (6)	27 (19)	139 (100)
Rubber additives	2342	4 (12)	11 (32)	3 (9)	9 (26)	0 (0)	2 (6)	5 (15)	34 (100)
Dyes	763	2 (7)	5 (17)	0 (0)	8 (28)	0 (0)	3 (10)	11 (38)	29 (100)
Topicals	2747	5 (20)	8 (32)	2 (8)	5 (20)	0 (0)	1 (4)	4 (16)	25 (100)
Corticosteroids	1178	2 (20)	3 (30)	0 (0)	3 (30)	0 (0)	0 (0)	2 (20)	10 (100)
Resins	1157	6 (20)	3 (10)	0 (3)	9 (30)	0 (0)	3 (10)	9 (30)	31 (100)
Total (excluding 0‐0‐0)	‐	61	152	14	153	1	43	129	553

*Note*: In brackets, the proportions of positive reactions divided by the total number of positive reactions for that allergen group on a particular day are given as percentages.

^a^ 0‐0‐0, no positive reaction on any day; 0‐0‐1, positive reaction on Day 7; 0‐1‐1, positive reactions on Days 3 and 7, and so on.

^b^ Absolute numbers and proportions of positive reactions on certain reading days observed in groups may deviate from absolute numbers and proportion observed in particular allergens belonging to that group, for example diazolinidyl urea.

Out of 553 positive reactions, 148 (26.8%) reactions were considered clinically relevant, observed in 76 (19.2%) patients. Relevant isolated D7 reactions were particularly noted in the group of fragrances (Table S4). Relevant isolated positive reactions were seen on D2, D3 and D7 in 0 (0%), 12 (2.2%) and 9 (1.6%) occasions, observed in 0 (0%), 11 (2.8%) and 9 (2.3%) patients, respectively (data not shown). The proportion of relevant reactions out of the total number of positive reactions were 0%, 7.9% and 14.8% on D2, D3 and D7, respectively.

### 3.2 Factors that may be associated with isolated D7 reaction

A summary of patient characteristics is given in Table 2. Of note is the high percentage of females (80.3%) and the preponderance of the head (62.9%) as the main affected body location.

**TABLE 2:** Patient characteristics

Variable	*N* = 396^a^
Sex	
Male	78 (19.7%)
Female	318 (80.3%)
Age, category	
<18	24 (6.1%)
18–30	78 (19.7%)
31–45	99 (25.0%)
46–60	111 (28.0%)
>60	84 (21.2%)
History of atopic dermatitis	
No	218 (67.5%)
Yes	105 (32.5%)
Unknown	73
Main body location	
Head	249 (62.9%)
Hands	65 (16.4%)
Feet	13 (3.3%)
Other	69 (17.4%)

^a^ Statistics presented: *n* (%).

Univariable regression analysis showed that the factors sex, past or current atopic dermatitis, and body location did not predict isolated D7 reactions (Table 3). Significant associations were found for age (OR 1.02, 95% CI 1.00–1.04), metals (OR 12.9, 95% CI 5.50–30.4), fragrances (OR 3.66, 95% CI 1.38–9.72) and resins (OR 2.81, 95% CI 1.06–7.47).

**TABLE 3:** Mixed model logistic regression analysis in 51 patients with isolated positive reaction on Day 7 as dependent variable

Variable	Isolated reaction on Day 7		Univariable			Multivariable		
	No, *N* = 14 986^a^	Yes, *N* = 61	OR	95% CI	*p*‐value	OR	95% CI	*p*‐value
Sex					0.64			
Male	2953 (19.7%)	10 (16.4%)	—^b^	—				
Female	12 033 (80.3%)	51 (83.6%)	1.23	0.51, 2.93				
Age, category								
<18	911 (6.1%)	1 (1.6%)	—	—				
18–30	2957 (19.7%)	6 (9.8%)	1.71	0.17–16.9				
31–45	3748 (25.0%)	14 (23.0%)	3.17	0.35–28.4				
46–60	4191 (28.0%)	27 (44.3%)	5.52	0.64–47.7				
>60	3179 (21.2%)	13 (21.3%)	3.56	0.39–32.2				
Age (year)^c^	45 (30–59)^d^	54 (42–59)	1.02	1.00–1.04	0.048	1.02^e^	1.00–1.04	0.049
History of atopic dermatitis					0.22			
No^f^	11 016 (73.5%)	42 (68.9%)	—	—				
Yes	3970 (26.5%)	19 (31.1%)	1.20	0.58–2.49				
Main body location					0.75			
Head	9420 (62.9%)	42 (68.9%)	—	—				
Other^g^	2615 (17.4%)	7 (11.5%)	0.63	0.24–1.69				
Hands	2458 (16.4%)	11 (18.0%)	1.01	0.42–2.44				
Feet	493 (3.3%)	1 (1.6%)	0.48	0.05–4.90				
Allergen Group^h^					<0.001			<0.001
Preservatives	3952 (26.4%)	7 (11.5%)	—	—			—^i^	
Fragrances	1574 (10.5%)	10 (16.4%)	3.66	1.38–9.72		3.53	1.53–8.10	
Metals	1163 (7.8%)	25 (41.0%)	12.9	5.50–30.4		12.5	6.29–24.7	
Rubber additives	2372 (15.8%)	4 (6.6%)	0.96	0.28–3.29				
Dyes	790 (5.3%)	2 (3.3%)	1.45	0.30–7.03				
Topical agents	2767 (18.5%)	5 (8.2%)	1.03	0.32–3.26		0.99	0.35–2.78	
Corticosteroids	1186 (7.9%)	2 (3.3%)	0.95	0.20–4.65		0.92	0.21–4.11	
Resins	1182 (7.9%)	6 (9.8%)	2.81	1.06–7.47		2.81	1.06–7.44	

*Note*: Independent variables related to isolated reaction on Day 7 described in terms of patch test reaction.

Abbreviations: CI, confidence interval; OR, odds ratio.

^a^ Statistics presented: *n* (%).

^b^ — denotes reference category.

^c^ ‘Age’ as continuous, linear variable; odds ratio is per year.

^d^ Median value and interquartile range.

^e^ The independent factors age, ‘relevance’, fragrances, metals and resins were used in the multivariable logistic regression model. No interactions between these independent factors were noted.

^f^ Categories ‘no’ and ‘unknown’ are taken together.

^g^ In order of frequency: trunk, arms, legs, entire body, armpits, groins, buttocks, genitals.

^h^ Patients could have more than one positive reaction per allergen group, for example, if cobalt and nickel both were positive in a patient it was analysed as one reaction.

^i^ For reference category ‘others’ (preservatives, rubber additives and dyes) were taken.

## 4 DISCUSSION

### 4.1 Occurrence of isolated D7 reactions

The first purpose of this study was to investigate the value of performing an additional D7 patch test reading at a large secondary referral centre. To our knowledge, this is the first study on this topic. The overall prevalence figures for positive reactions to allergens on any day mirror those in the European population.^10^ The lower figures for methylisothiazolinone in our study may be explained by the fact that this agent was included during the last part of the study period.

Out of the 553 positive reactions, 61 reactions were exclusively positive on D7, observed in 51 patients. Our study corroborates findings in earlier studies in which isolated D7 positive reactions were described in 4%–30% of the patients.^2^, ^3^, ^4^, ^5^, ^6^, ^7^, ^8^, ^9^ Our study has demonstrated the highest proportions of isolated late reactions for topicals, corticosteroids and resins. Neomycin is the most cited contact allergen for its potential to induce late reactions,^2^, ^3^, ^4^, ^5^, ^6^, ^7^, ^9^, ^14^ followed by corticosteroids.^1^, ^2^, ^3^, ^6^, ^7^, ^14^ Metals were also involved in late reactions.^3^, ^4^, ^5^, ^7^, ^8^, ^9^, ^14^, ^15^ Other ‘late’ allergens mentioned in the literature were preservatives,^2^, ^3^, ^4^, ^7^, ^8^ colophony,^4^, ^8^ fragrances,^3^, ^8^ p‐phenylenediamine,^2^, ^5^ epoxy resin,^2^, ^3^ and acrylates.^15^ Our investigation confirms the results of a recent large Dutch study on the value of late reading in which the most notable late reactors in order of proportion frequency were topicals, corticosteroids, dyes, fragrances, metals and preservatives.^3^

Our study has demonstrated a low percentage of isolated positive D2 readings as opposed to isolated positive D7 readings. These findings agree with those of previous studies that described the low frequency of early reactions.^4^, ^5^

Clinical relevance of a positive reaction is important given the fact that an extra reading should be of practical advantage. Relevant late positive reactions were found in 28 out of the 34 late reactions in the study by McFarlane et al.^4^ In the current study 9 out 61 positive isolated D7 reactions were deemed relevant in 9 out of the 51 patients having this late reaction. The lower number of relevant reactions in our study may be caused by the fact that our population is less selected. Relevant isolated late positive reactions in our study were particularly seen in the group of fragrances.

The assessment of clinical relevance is an important tool in the interpretation of positive patch test reactions.^1^ However, this may be difficult in clinical practice. In the current study, it was based on the observation that the dermatitis disappeared after cessation of contact with the suspected allergen and/or on the patient's history. The label of ingredients of cosmetic products should be checked or spot test should be performed.^1^ However, in the current study ingredients of cosmetics were not checked in all cases, and no spot tests were done. Furthermore, it was not possible to make the subdivision into ‘past’ and ‘unknown’ relevance in many cases.

Notwithstanding these drawbacks, we are convinced of the reliability of clinical relevance because it was obvious from the patient's history that the majority of these reactions were of past or unknown relevance and were not contributive to their current dermatitis.

Based on the abovementioned considerations, we advise to omit the D2 reading in a selected patient group on strict conditions. Important considerations are that the baseline series is tested only, without additional test series, in patients who are able to follow the clear instructions on how to remove the patch test.

### 4.2 Factors that may be associated with isolated D7 reaction

The second aim of this study was to determine the factors that were associated with a reading exclusively positive on D7. On the one hand, it was demonstrated that the factors sex, past or current atopic dermatitis, and body location did not predict late reactions. On the other hand, significant associations were found for age and the allergen groups of metals, fragrances and resins.

Our results confirmed the lack of influence of atopic dermatitis on late positive readings reported in earlier studies.^3^, ^16^ In line with two investigations, our results pointed to a predictive capacity of higher age.^2^, ^3^ In elderly individuals, reduced rates of thymic lymphocyte proliferation were described, which in turn resulted in reduced numbers of lymphocytes in periferal lymphoid tissues.^17^ Together with other immunological changes, this caused a diminished immune function.^17^ These factors may account for a slower progress of the elicitation phase of the allergic contact reaction in elderly patients.

Previous studies have found female gender,^2^ and the allergen groups of topicals and corticosteroids^3^ to be predictors of late reactions. The late reaction on corticosteroids may be explained by their anti‐inflammatory effect.^3^ With regard to allergen group, the current study showed similar patterns, although at a lower level. Our proportion scores were highest for topicals, corticosteroids and resins, and the absolute scores were highest for metals and fragrances. Explanations for the fact that we did not find topicals and corticosteroids as predictive factors in the regression analyses may be the fact that in our study the variables were described in terms of reaction instead of patients, and the lower absolute numbers of positive test results for topicals and corticosteroids. This in turn may be caused by a different case mix in our secondary centre population as compared with the tertiary centre population in the recent Dutch study.^3^ Striking features in our investigation were the high percentage of females and the preponderance of the head as the affected body site. A considerable part of our study population was composed of females with a history of atopic dermatitis having current facial dermatitis who were tested to rule out contact allergy. Schnuch et al. have found an increased proportion of females and history of atopic dermatitis in facial dermatitis.^18^ Nickel and cosmetic allergens, including fragrance, were significantly more common in women than men.^18^ These features may bias the selection process, which may cause the different pattern of predictive allergen groups observed in our study as opposed to the previous study.^3^ Furthermore, our different case mix may potentially account for the lack of gender and body location as predictive factors for late reactions. These aberant features may indicate the different nature of a secondary referral centre.

### 4.3 Strength and limitations

A novel aspect was the fact that this study proved the value of a late reading at a secondary referral centre, with its different case mix, whereas all previous studies were done at tertiary centres.

For the first time body location was used as a predictive factor for late positive readings.

Limitations are the relatively small population size and the fact that methylisothiazolinone was tested in only a small group, which could have influenced the outcomes particularly with respect to the regression analysis. Other limitations are the retrospective nature of the study, the lack of strict criteria for defining clinical relevance, and the lack of division into past and unknown relevance.

## 5 CONCLUSIONS

In this study, it was found that D7 scoring should be routinely adopted at secondary referral centres. It was also shown that higher age, and the allergen groups of metals, fragrances and resins could predict late reactions.

## CONFLICT OF INTEREST

The authors report no conflicts of interest.

## AUTHOR CONTRIBUTIONS

**R. A.** **Tupker:** Conceptualization; Data curation; Formal analysis; Investigation; Methodology; Project administration; Software; Supervision; Writing – original draft; Writing – review & editing. **W. G. C.** **Stapper:** Conceptualization; Data curation; Formal analysis; Investigation; Methodology; Project administration; Supervision; Writing – original draft; Writing – review & editing. **J. C.** **Kelder:** Conceptualization; Data curation; Formal analysis; Investigation; Methodology; Project administration; Supervision; Writing – original draft; Writing – review & editing.

## Supporting information

Supporting Information S1

## Data Availability

The data that support the findings of this study are available from the corresponding author upon reasonable request.
