# Supplementary material for: Predictive factors for Day 7 positive patch test readings at a secondary referral centre
Source: Skin Health Dis. 2021 Nov 24;2(1):e79. doi: 10.1002/ski2.79 (PMC9060153; doi:10.1002/ski2.79)
Supplement: Supplementary file 1 — Supporting Information S1 [file SKI2-2-e79-s001.docx]

Table S1. Distribution of patient numbers and reaction numbers over the timing of reaction, according to the permutation scheme.

| timing of reaction | # patients | # reactions |
| --- | --- | --- |
| 0 - 0 - 0^a^ | 396 | 14492 |
| 0 - 0 - 1 | 51 | 61 |
| 0 - 1 - 0 | 108 | 152 |
| 1 - 0 - 0 | 10 | 14 |
| 0 - 1 - 1 | 105 | 153 |
| 1 - 0 - 1 | 1 | 1 |
| 1 - 1 - 0 | 29 | 43 |
| 1 - 1 - 1 | 92 | 129 |
| total (excluding 0-0-0) | 396^b^ | 553 |

^a^ 0-0-0, no positive reaction on any day; 0-0-1, positive reaction on day 7; 0-1-1, positive reactions on days 3 and 7, etc.

^b^ Some patients had an isolated positive reaction (e.g. 0-0-1) combined with a positive reaction to another allergen on another day. The total number of patients is thus higher than the sum of the number for each timing group.

Table S2. Groups of allergens.

| Allergen group | allergen |
| --- | --- |
|  |  |
| Metals | Potassium dichromate |
|  | Cobalt chloride |
|  | Nickel sulphate |
|  |  |
| Preservatives | Formaldehyde |
|  | Paraben mix |
|  | Quaternium-15 |
|  | Methylchloroisothiazolinone |
|  | Methylisothiazolinone |
|  | Methyldibromo glutaronitrile |
|  | Thimerosal |
|  | Ethylenediamine dihydrochloride |
|  | Diazolidinyl urea |
|  | Imidazolidinylurea |
|  |  |
| Fragrances | Myroxylon pereirae (balsam of Peru) |
|  | Fragrance mix I |
|  | Hydroxyisohexyl 3-cyclohexane carboxaldehyde (HICC) (Lyral) |
|  | Fragrance mix II |
|  |  |
| Rubber additives | Thiuram mix |
|  | N-isopropyl-N-phenyl-para-phenylenediamine (IPPD) |
|  | Mercapto mix |
|  | Mercaptobenzothiazole |
|  | Black rubber mix |
|  | Carba mix |
|  |  |
| Dyes | Para-phenylenediamine (PPD) |
|  | Textile dye mix |
|  |  |
| Topicals | Neomycin sulphate |
|  | Benzocaine |
|  | Lanolin alcohols |
|  | Sesquiterpene lactone mix |
|  | Caine mix IV |
|  | Quinoline mix |
|  |  |
| Corticosteroids | Budesonide |
|  | Tixocortol pivalate |
|  | Hydrocortisone-17-butyrate |
|  |  |
| Resins | Colophony |
|  | Epoxy resin |
|  | p-tert-butylphenol formaldehyde resin |

Table S3. Patch test results obtained with the allergens of the European extended baseline series on days 2, 3 and 7 according to the permutation scheme. In brackets the figures for proportions are given, i.e., the number of positive reactions on a given day divided by the total number of positive reactions for that allergen.

| Allergen | 0 - 0 - 0^a^ | 0 - 0 - 1 | 0 - 1 - 0 | 1 - 0 - 0 | 0 - 1 - 1 | 1 - 0 - 1 | 1 - 1 - 0 | 1 - 1 - 1 | day 2, 3 and/or 7 |
| --- | --- | --- | --- | --- | --- | --- | --- | --- | --- |
| Benzocaine 5% pet | 396 | 0 (0) | 0 (0) | 0 (0) | 0 (0) | 0 (0) | 0 (0) | 0 (0) | 0 (100) |
| Black rubber mix 0.6% pet | 390 | 1 (17) | 0 (0) | 0 (0) | 2 (33) | 0 (0) | 1 (17) | 2 (33) | 6 (100) |
| Budesonide 0.01% pet | 391 | 1 (20) | 2 (40) | 0 (0) | 2 (40) | 0 (0) | 0 (0) | 1 (33) | 5 (100) |
| Caine mix IV 10% pet | 392 | 0 (0) | 1 (25) | 1 (25) | 1 (25) | 0 (0) | 0 (0) | 1 (25) | 4 (100) |
| Carba mix 3% pet | 381 | 2 (13) | 8 (53) | 1 (7) | 3 (20) | 0 (0) | 1 (7) | 0 (0) | 15 (100) |
| Clioquinol 5% pet | 396 | 0 (0) | 0 (0) | 0 (0) | 0 (0) | 0 (0) | 0 (0) | 0 (0) | 0 (100) |
| Cobalt chloride 1% pet | 365 | 7 (23) | 7 (23) | 0 (0) | 8 (26) | 0 (0) | 3 (10) | 6 (19) | 31 (100) |
| Colophony 20% pet | 378 | 2 (11) | 2 (11) | 0 (0) | 5 (28) | 0 (0) | 3 (17) | 6 (33) | 18 (100) |
| Diazolinidyl urea 2% pet | 395 | 1 (100) | 0 (0) | 0 (0) | 0 (0) | 0 (0) | 0 (0) | 0 (0) | 1 (100) |
| Epoxy resin 1% pet | 388 | 4 (50) | 0 (0) | 0 (0) | 2 (25) | 0 (0) | 0 (0) | 2 (25) | 8 (100) |
| Ethylenediamine 1% pet | 390 | 2 (33) | 3 (50) | 0 (0) | 1 (17) | 0 (0) | 0 (0) | 0 (0) | 6 (100) |
| Formaldehyde 2% aq | 381 | 1 (7) | 5 (33) | 1 (7) | 6 (40) | 0 (0) | 1 (7) | 1 (7) | 15 (100) |
| Fragrance mix I 8% pet | 349 | 4 (9) | 19 (40) | 1 (2) | 10 (21) | 0 (0) | 4 (9) | 9 (19) | 47 (100) |
| Fragrance mix II 14% pet | 365 | 1 (3) | 10 (32) | 1 (3) | 11 (35) | 1 (3) | 1 (3) | 6 (19) | 31 (100) |
| Hydrocortisone-17-butyrate 1% pet | 394 | 1 (50) | 0 (0) | 0 (0) | 1 (50 | 0 (0) | 0 (0) | 0 (0) | 2 (100) |
| Hydroxyisohexyl 3-cyclohexane carboxaldehyde (HICC) (Lyral) 5% pet | 386 | 2 (20) | 1 (10) | 0 (0) | 4 (40) | 0 (0) | 1 (10) | 2 (20) | 10 (100) |
| Imidazolidinyl urea 2% aq | 395 | 0 (0) | 1 (100) | 0 (0) | 0 (0) | 0 (0) | 0 (0) | 0 (0) | 1 (100) |
| Lanolin alcohols 30% pet | 382 | 1 (7) | 7 (50) | 1 (7) | 4 (29) | 0 (0) | 1 (7) | 0 (0) | 14 (100) |
| Mercapto mix 2% pet | 392 | 0 (0) | 1 (25) | 1 (25) | 1 (25) | 0 (0) | 0 (0) | 1 (25) | 4 (100) |
| 2-Mercaptobenzothiazole 2% pet | 394 | 0 (0) | 1 (50) | 0 (0) | 1 (50) | 0 (0) | 0 (0) | 0 (0) | 2 (100) |
| Methyldibromoglutaronitrile 0.3% pet | 377 | 1 (5) | 12 (63) | 1 (5) | 2 (11) | 0 (0) | 1 (5) | 2 (11) | 19 (100) |
| Methylisothiazolinone + methylchloroisothiazolinone 0.02% aq | 367 | 2 (7) | 9 (31) | 0 (0) | 9 (31) | 0 (0) | 3 (10) | 6 (21) | 29 (100) |
| Methylisothiazolinone 0.2% aq | 57 | 0 (0) | 0 (0) | 0 (0) | 2 (67) | 0 (0) | 0 (0) | 1 (33) | 3 (100) |
| Myroxylon pereirae (balsam of Peru) 25% pet | 345 | 3 (6) | 18 (35) | 2 (4) | 16 (31) | 0 (0) | 2 (4) | 10 (20) | 51 (100) |
| N-isopropyl-N-phenyl-para-phenylenediamine (IPPD) 0.1% pet | 390 | 1 (17) | 1 (17) | 0 | 2 (33) | 0 (0) | 0 (0) | 2 (33) | 6 (100) |
| Neomycin sulphate 20% pet | 392 | 4 (100) | 0 (0) | 0 (0) | 0 (0) | 0 (0) | 0 (0) | 0 (0) | 4 (100) |
| Nickel sulphate 2.5% pet | 274 | 12 (10) | 15 (12) | 2 (2) | 37 (30) | 0 (0) | 11 (9) | 45 (37) | 122 (100) |
| p-phenylenediamine 1% pet | 371 | 2 (8) | 5 (20) | 0 (0) | 5 (20) | 0 (0) | 2 (8) | 11 (44) | 25 (100) |
| p-tert-butylphenol formaldehyde resin 1% pet | 392 | 0 (0) | 1 (25) | 0 (0) | 2 (50) | 0 (0) | 0 (0) | 1 (25) | 4 (100) |
| Paraben mix 16% pet | 392 | 0 (0) | 4 (100) | 0 (0) | 0 (0) | 0 (0) | 0 (0) | 0 (0) | 4 (100) |
| Potassium dichromate 0.5% pet | 351 | 6 (13) | 15 (33) | 1 (2) | 10 (22) | 0 (0) | 5 (11) | 8 (18) | 45 (100) |
| Primin 0.01% pet | 392 | 0 (0) | 0 (0) | 0 (0) | 3 (75) | 0 (0) | 1 (25) | 0 (0) | 4 (100) |
| Quaternium-15 1% pet | 391 | 0 (0) | 1 (20) | 0 (0) | 1 (20) | 0 (0) | 1 (20) | 2 (40) | 5 (100) |
| Quinoline mix 6% pet | 395 | 0 (0) | 0 (0) | 0 (0) | 0 (0) | 0 (0) | 0 (0) | 1 (100) | 1 (100) |
| Sesquiterpene lactone mix 0.1% pet | 394 | 0 (0) | 0 (0) | 0 (0) | 0 (0) | 0 (0) | 0 (0) | 2 (100) | 2 (100) |
| Thimerosal 0.1% pet | 391 | 0 (0) | 2 (40) | 0 (0) | 2 (40) | 0 (0) | 1 (20) | 0 (0) | 5 (100) |
| Thiuram mix 1% pet | 395 | 0 (0) | 0 (0) | 1 (100) | 0 (0) | 0 (0) | 0 (0) | 0 (0) | 1 (100) |
| Tixocortol pivalate 0.1% pet | 393 | 0 (0) | 1 (33) | 0 (0) | 0 (0) | 0 (0) | 0 (0) | 2 (67) | 3 (100) |

^a^ 0-0-0, no positive reaction on any day; 0-0-1, positive reaction on day 7; 0-1-1, positive reactions on days 3 and 7, etc.

Table S4. Patch test results of number of positive reactions with current relevance in the allergen groups on days 2, 3 and 7 according to the permutation scheme.

| Allergen group | 0 - 0 - 0^a^ | 0 - 0 - 1 | 0 - 1 - 0 | 1 - 0 - 0 | 0 - 1 - 1 | 1 - 0 - 1 | 1 - 1 - 0 | 1 - 1 - 1 | day 2, 3, 7 |
| --- | --- | --- | --- | --- | --- | --- | --- | --- | --- |
| Metals |  | 2 | 3 | 0 | 5 | 0 | 3 | 20 | 33 |
| Preservatives |  | 1 | 1 | 0 | 2 | 0 | 0 | 4 | 8 |
| Fragrances |  | 5 | 3 | 0 | 16 | 0 | 3 | 33 | 60 |
| Rubber additives |  | 0 | 2 | 0 | 2 | 0 | 0 | 4 | 8 |
| Dyes |  | 0 | 2 | 0 | 1 | 0 | 2 | 11 | 16 |
| Topicals |  | 0 | 1 | 0 | 2 | 0 | 1 | 5 | 9 |
| Corticosteroids |  | 1 | 0 | 0 | 3 | 0 | 0 | 4 | 8 |
| Resins |  | 0 | 0 | 0 | 1 | 0 | 0 | 5 | 6 |
| Total |  | 9 | 12 | 0 | 32 | 0 | 9 | 86 | 148 |

^a^ 0-0-0, no positive reaction on any day; 0-0-1, positive reaction on day 7; 0-1-1, positive reactions on days 3 and 7, etc.
